# Optical image and Vickers hardness dataset for repair of 1080 steel using additive friction stir deposition of Aermet 100

**DOI:** 10.1016/j.dib.2022.107862

**Published:** 2022-01-22

**Authors:** Kathleen Chou, Michael Eff, Chase D. Cox, Connor Saukas, Jason Carroll

**Affiliations:** a Center for Materials and Manufacturing, Eaton Corporation, Southfield, MI 48076, USA; b EWI, Columbus, OH 43221, USA; c MELD Manufacturing, Christiansburg, VA 24073, USA

**Keywords:** Solid-state additive manufacturing, Repair, Additive friction stir deposition, Severe plastic deformation

## Abstract

This article presents optical images, measurements of heat affected zone depths, and peak Vickers hardness values from the heat affected zone regions of a 1080 steel plate substrate with a machined groove “flaw” repaired using additive friction stir deposition of Aermet 100. The deposition of Aermet 100 was performed using a L3 Meld machine with 9.525 mm (0.375 inch) square bar profile Aermet 100 feedstock rods fed through a hollow, 10.16 mm (0.4 inch) diameter, rotating tool onto a 1.02 mm thick 1080 steel plate with a machined groove “flaw” along the plate's length and bisecting the plate's width. The depth of the machined groove “flaw” ranged from 4.7625, 6.35, and 9.525 mm. The data is categorized into four groups: multi-layer builds deposited at room temperature with and without a cooling plate, 2-layer builds deposited at room temperature without a cooling plate, single-layer builds deposited at room temperature without a cooling plate, and a design of experiments for single-layer builds that varied the spindle rotation speed (RPM), travel speed (mm/min), material feed rate (mm/min), and pre-heat temperature (°C) of the deposition. The data shows the parameter conditions that achieved flaw-free consolidated repairs and the associated depth and peak Vickers hardness of the heat affected zone. Optical images of cross-sectioned deposition regions were obtained using an optical microscope with Leco Olympus DP27 macro camera, and Vickers hardness line traces were measured along the depth of the deposited and heat affected zone extending into the substrate on cross-sectioned samples using a Leco LM 247AT microhardness tester. The depth of the heat affected zone is reported as the measured average of five individual data points. Peak values for Vickers hardness for the heat affected zone decreased for pre-heated conditions at 329°C compared to builds conducted at room temperature of 21°C. This dataset provides visual characterization and associated hardness measurements of as-deposited repairs of dissimilar steel alloys. This article can be used to inform parameter selection for additive friction stir deposition of dissimilar steel materials for repair and solid-state additive manufacturing applications.

## Specifications Table

**Table:** 

Subject	Metals and Alloys
Specific subject area	Solid-state additive manufacturing
Type of data	Image Table
How the data were acquired	Optical images were captured using an optical microscope with Leco Olympus DP27 macro camera. The depth of the heat affected zone is reported as the average of five individual data points measured using ImageJ image processing program. Microhardness testing was performed using Leco LM 247AT microhardness tester microhardness tester tested at 1000 gf (9.81 N) load and with a 13 s dwell time. Hardness traces were measured on polished cross sections with a 0.50 mm spacing between indents along the depth of the deposited and heat affected zone extending into the substrate on cross-sectioned samples.
Data format	Raw data
Description of data collection	Optical images, heat affected zone depths, and Vickers microhardness data were collected in four groups: multi-layer builds, 2-layer builds, single-layer builds, and a design of experiments for single-layer builds. The deposition conditions for each condition are described in Experimental design section.
Data source location	Material Deposition: MELD Manufacturing, Christiansburg, Virginia 24073, USA Optical Images, Heat Affected Zone Depth Measurements, and Vickers Hardness Values: EWI, Columbus, OH 43221, USA
Data accessibility	With the article

## Value of the Data

• This dataset containing optical images, heat affected zone depth measurements, and Vickers hardness measurements from the heat affected zone regions of additive friction stir deposition of Aermet 100 on a 1080 steel plate substrate is useful for insight on suitable parameter conditions for flaw-free consolidated repairs during additive friction stir deposition of dissimilar steel alloys.
• This image, heat affected zone depth, and hardness dataset is a useful resource for metallurgical, material, mechanical, and welding researchers and engineers.
• The dataset can be used to inform candidate parameter conditions for researchers developing experiments that investigate solid-state additive manufacturing processes such as additive friction stir deposition.

## 1 Data Description

The data images are categorized into four groups: multi-layer builds deposited at room temperature with and without a cooling plate, 2-layer builds deposited at room temperature without a cooling plate, single-layer builds deposited at room temperature without a cooling plate, and a design of experiments for single-layer builds that varied the spindle rotation speed (RPM), travel speed (mm/min), material feed rate (mm/min), and pre-heat temperature (°C) of the deposition. The specific parameter conditions for each group and trial are presented in Section 2. Each data image corresponds to a cross section of an as-deposited trial.

### 1.1 Group 1: multi-layer builds (MLB)

**Fig. 1:**
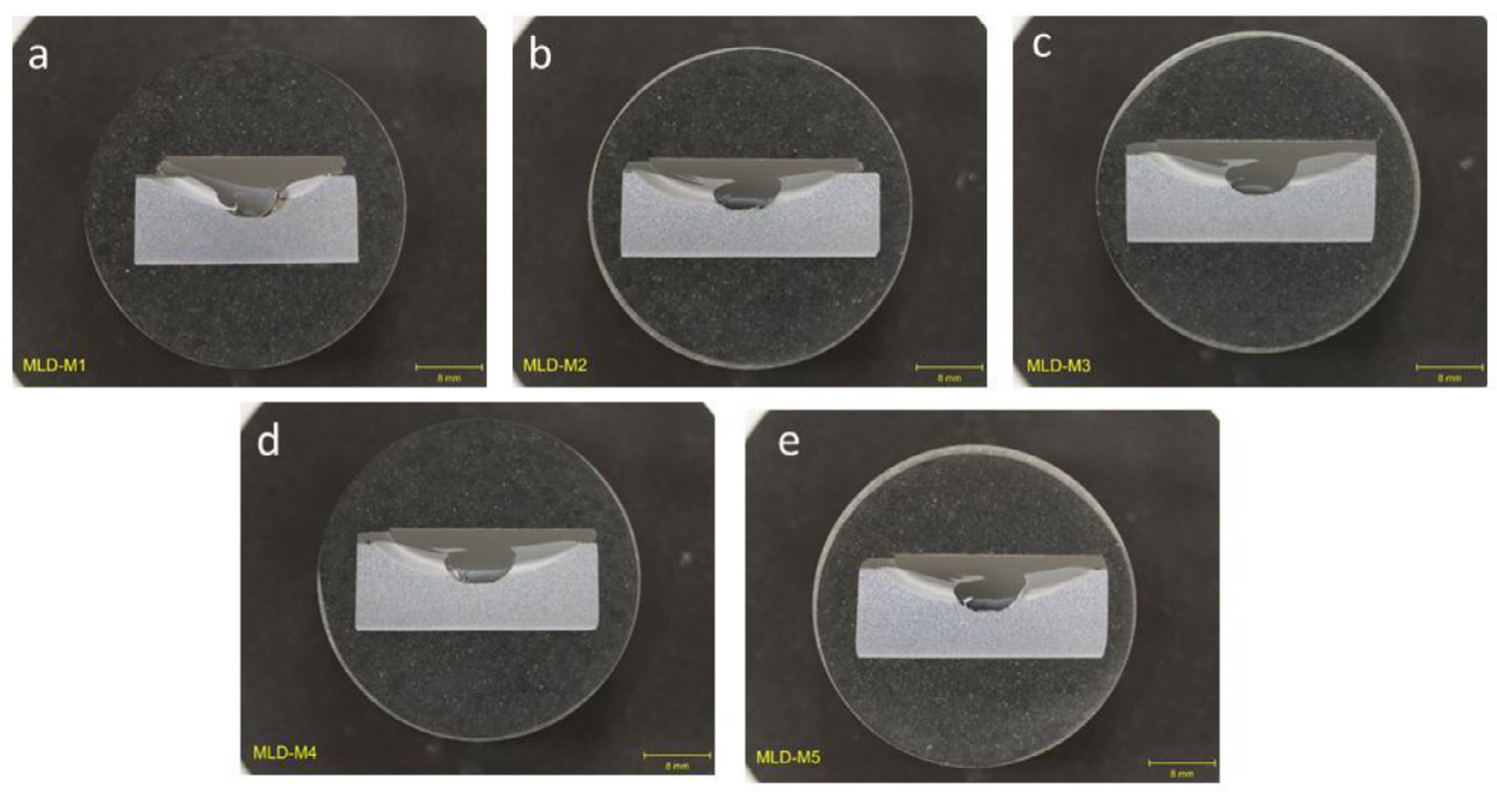
Optical images of cross-section for as-deposited trials in Group 1: MLB for multi-layer builds deposited at room temperature with and without a cooling plate, showing lack of bonding and unfilled regions in deposited region for all trials in Group 1. (a) MLD-M1. (b) MLD-M2. (c) MLD-M3. (d) MLD-M4. (e) MLD-M5.

Cross-sectional optical images for group that encompasses deposition parameters for multi-layer builds deposited at room temperature with and without a cooling plate (Fig. 1). All deposits in this group showed lack of bonding around the deposit and lack of complete filling of the flaw region.

### 1.2 Group 2: two-layer builds (TLB)

**Fig. 2:**
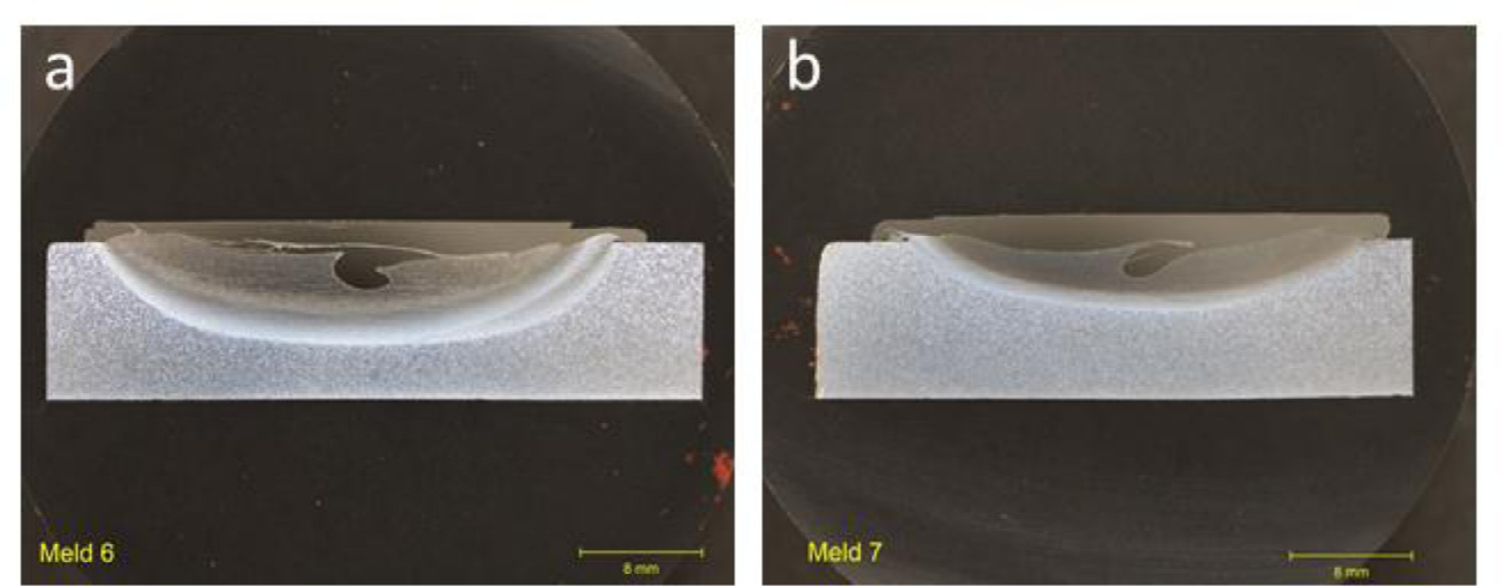
Optical images of cross-section for as-deposited trials in Group 2: TLB for 2-layer builds deposited at room temperature without a cooling plate showing complete filling and bonding of the deposited region. (a) Meld 6. (b) Meld 7.

Cross-sectional optical images for group that encompasses deposition parameters for 2-layer builds deposited at room temperature without a cooling plate (Fig. 2). All deposits in this group showed complete filling and bonding of the deposit with the substrate in the flaw region.

### 1.3 Group 3: single-layer builds (SLB)

**Fig. 3:**
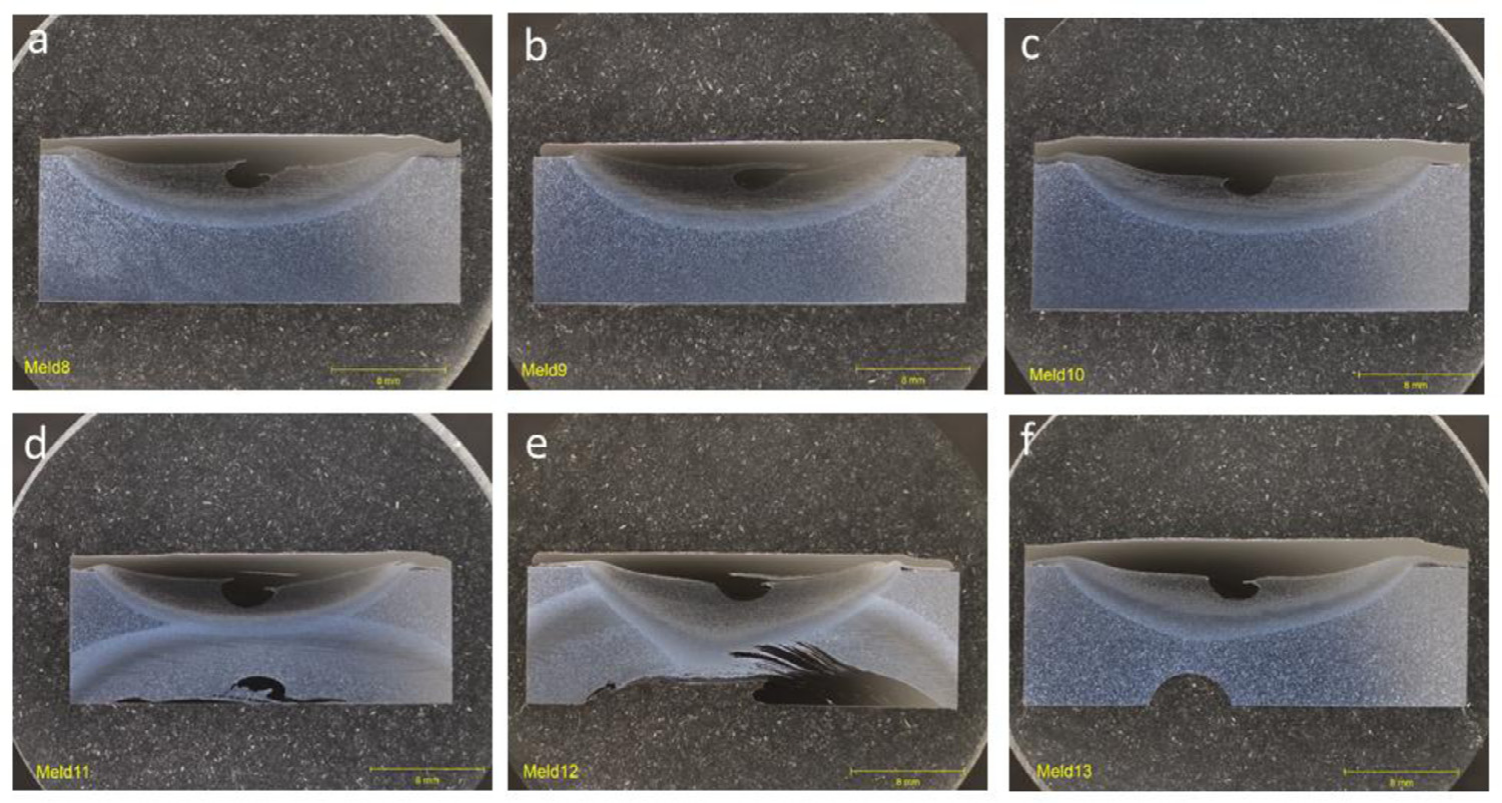
Optical images of cross-section for as-deposited trials in Group 3: SLB for single-layer builds deposited at room temperature without a cooling plate showing complete filling and bonding of the deposited region. (a) Meld 8. (b) Meld 9. (c) Meld 10. (d) Meld 11. (e) Meld 12. (f) Meld 13.

Cross-sectional optical images for group that encompasses deposition parameters for single-layer builds deposited at room temperature without a cooling plate (Fig. 3). All deposits in this group showed complete filling and bonding of the deposit with the substrate in the flaw region.

### 1.4 Group 4: design of experiments builds (DOE)

**Fig. 4:**
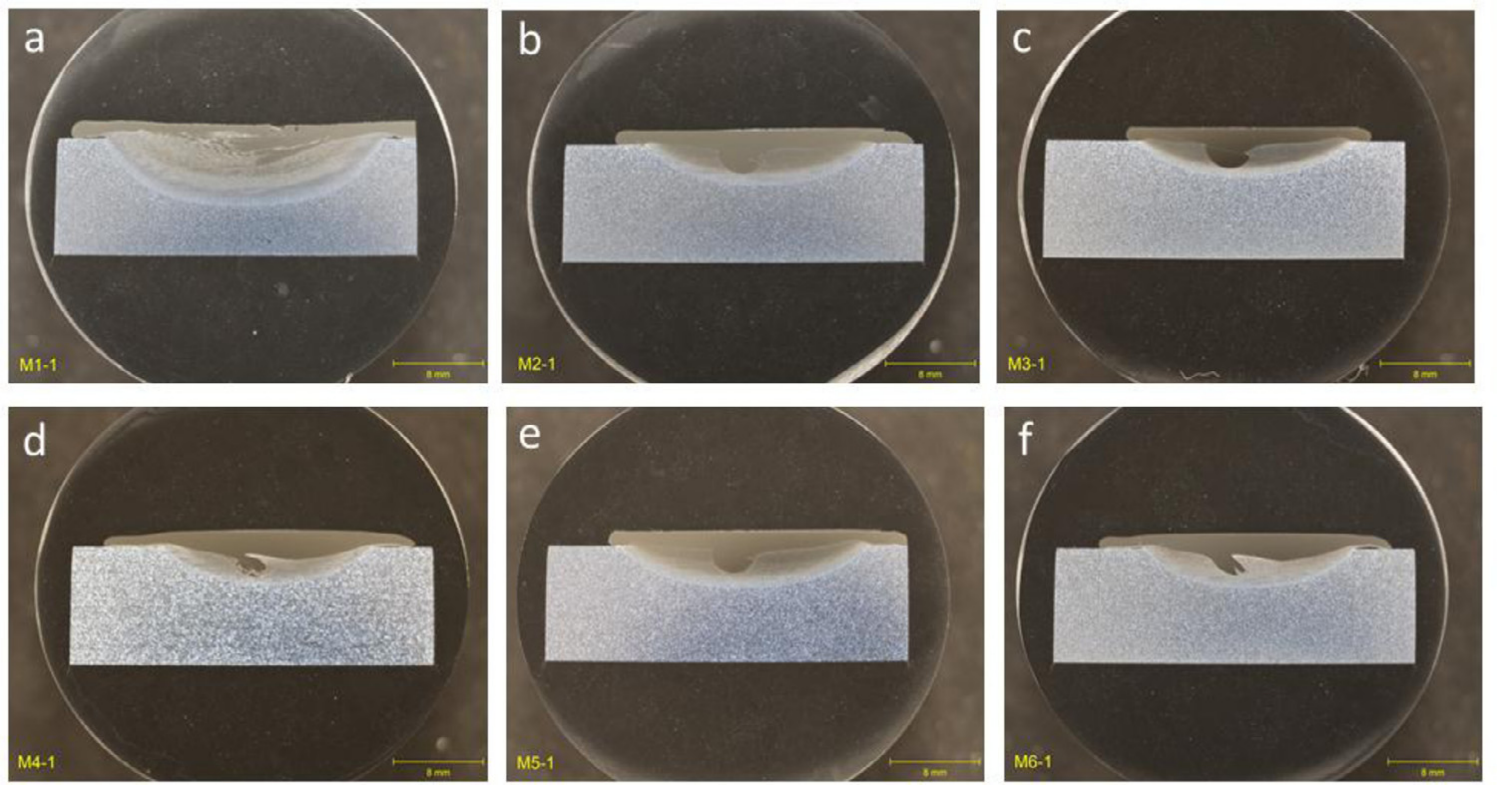
Optical images of cross-section for as-deposited trials in Group 4: DOE for single-layer builds that varied the spindle rotation speed (RPM), travel speed (RPM), and material feed rate (IPM) for a 21°C pre-heat temperature during the deposition. (a) M1-1. (b) M2-1. (c) M3-1. (d) M4-1. (e) M5-1. (f) M6-1.

**Fig. 5:**
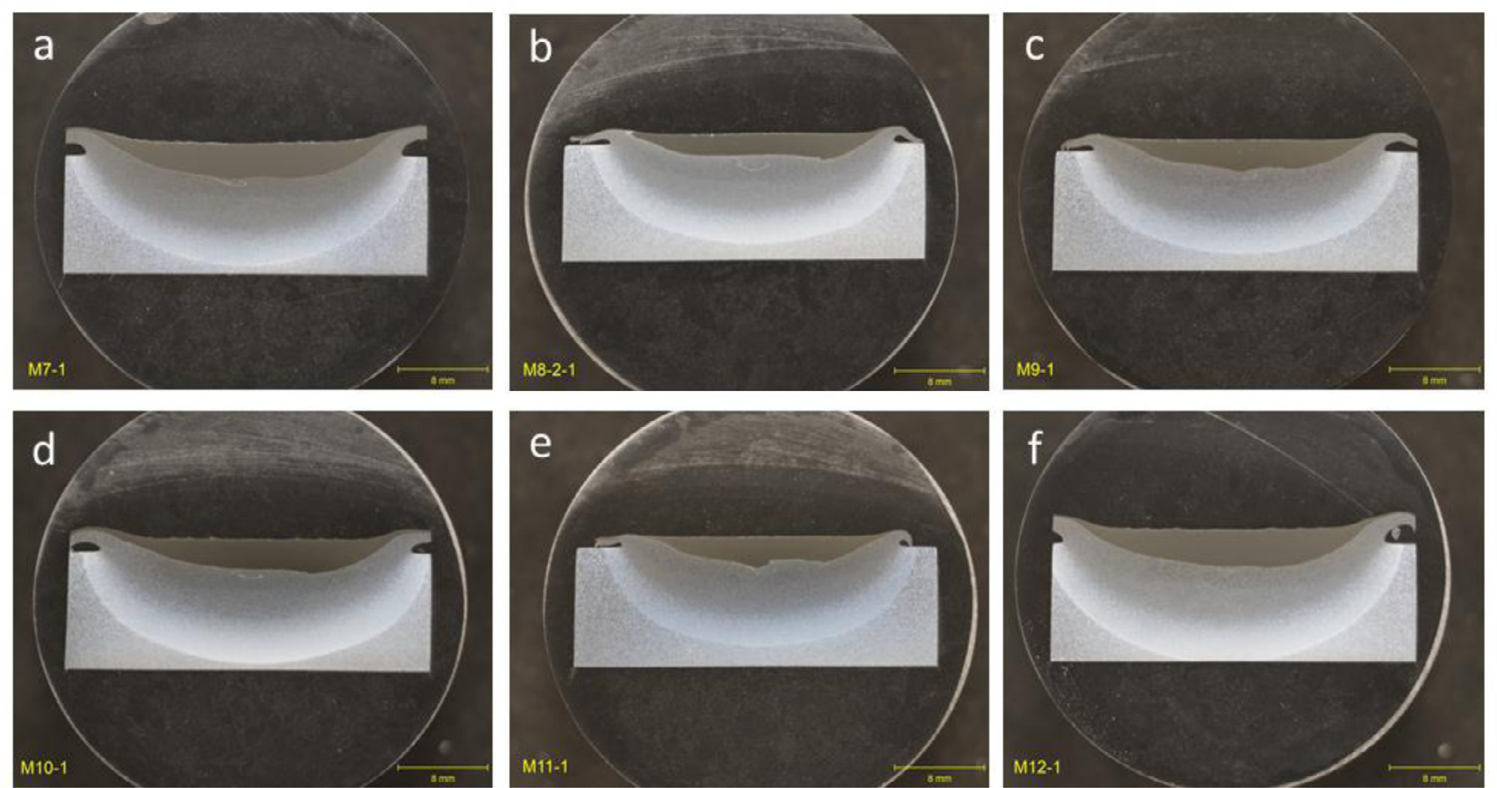
Optical images of cross-section for as-deposited trials in Group 4: DOE for single-layer builds that varied the spindle rotation speed (RPM), travel speed (RPM), and material feed rate (IPM) for a 329°C pre-heat temperature during the deposition. (a) M7-1. (b) M8-2-1. (c) M9-1. (d) M10-1. (e) M11-1. (f) M12-1.

Cross-sectional optical images for group that encompasses deposition parameters for design of experiments (DOE) for single-layer builds that varied the spindle rotation speed (RPM), travel speed (mm/min), material feed rate (mm/min), and pre-heat temperature (°C) of the deposition. Optical images for deposition trials performed at a pre-heat temperature of 21°C corresponding to room temperature are shown in Fig. 4. Optical images for deposition trials performed at a pre-heat temperature of 329°C corresponding to room temperature are shown in Fig. 5. All deposits in this group showed complete filling and bonding of the deposit with the substrate in the flaw region.

### 1.5 Depths of heat affected zone and peak Vickers hardness for fully bonded and filled deposit conditions

The depth of the heat affected zone is reported for deposition parameter conditions in Group 2, Group 3, and Group 4 that produced flaw-free deposits with complete filling and bonding of the deposit with the substrate in the flaw region is reported in Table 1. For Group 2 and Group 4, the peak Vickers hardness measured in the heat affected zone extending into the 1080 steel plate substrate on cross-sectioned samples is reported in Table 1. Vickers hardness data was not collected for Group 3 due to specimen geometry limitations. For Group 4, peak values for Vickers hardness for the heat affected zone decreased by approximately one-half for pre-heated conditions at 329°C compared to builds conducted at room temperature of 21°C.

**Table 1:** Depth of heat affected zone and peak Vickers hardness values in the heat affected zone for Group 2, Group 3, and Group 4 that produced deposits with complete filling and bonding of the deposit with the substrate in the flaw region.

Group Number	Specimen ID	Pre-heat temperature (°C)	Heat affected zone depths (mm)	Peak Vickers hardness (HV)
2: TLB	Meld 6	21	174.7	773
	Meld 7	21	120.5	832

3: SLB	Meld 8	21	130.4	-
	Meld 9	21	134.9	-
	Meld 10	21	127.9	-
	Meld 11	21	119.0	-
	Meld 12	21	144.8	-
	Meld 13	21	138.6	-

4: DOE	M1-1	21	153.7	768
	M2-1	21	93.9	824
	M3-1	21	81.3	825
	M4-1	21	85.0	654
	M5-1	21	95.5	823
	M6-1	21	89.9	675
	M7-1	329	247.1	372
	M8-2-1	329	226.2	372
	M9-1	329	234.3	364
	M10-1	329	251.4	402
	M11-1	329	224.6	387
	M12-1	329	261.1	369

## 2 Experimental Design, Materials and Methods

1080 rail steel plate (152.5 mm length, 127 mm width, 1.02 mm thickness) was sectioned using a band saw from a 136RE standard rail provided by Evraz [1], and a grooved “flaw” was machined into the 1080 steel plate bisecting the plate width and parallel to the plate's length. Additive friction stir deposition (AFSD) was used to deposit Aermet 100 (nominal composition in weight %: 0.21-0.25 C, 11-12 Ni, 13-14 Co, 2.9-3.3 Cr, 1.1-1.3 Mo, Balance Fe) in the machined flaw region as a repair method for the 1080 steel plate. AFSD is a solid-state additive manufacturing process in which feedstock material is passed through a hollow, non-consumable rotating tool head, and frictional heat is generated as the feed material and tool head contact the substrate [2,3]. The softened feed material is fed through the tool and metallurgically bonds with the substrate through high shear and severe plastic deformation at the interface [3]. AFSD has been demonstrated to produce site-specific buildup of fully-dense material in as-printed conditions with fine, equiaxed microstructures [4], [5], [6], [7], [8].

**Fig. 6:**
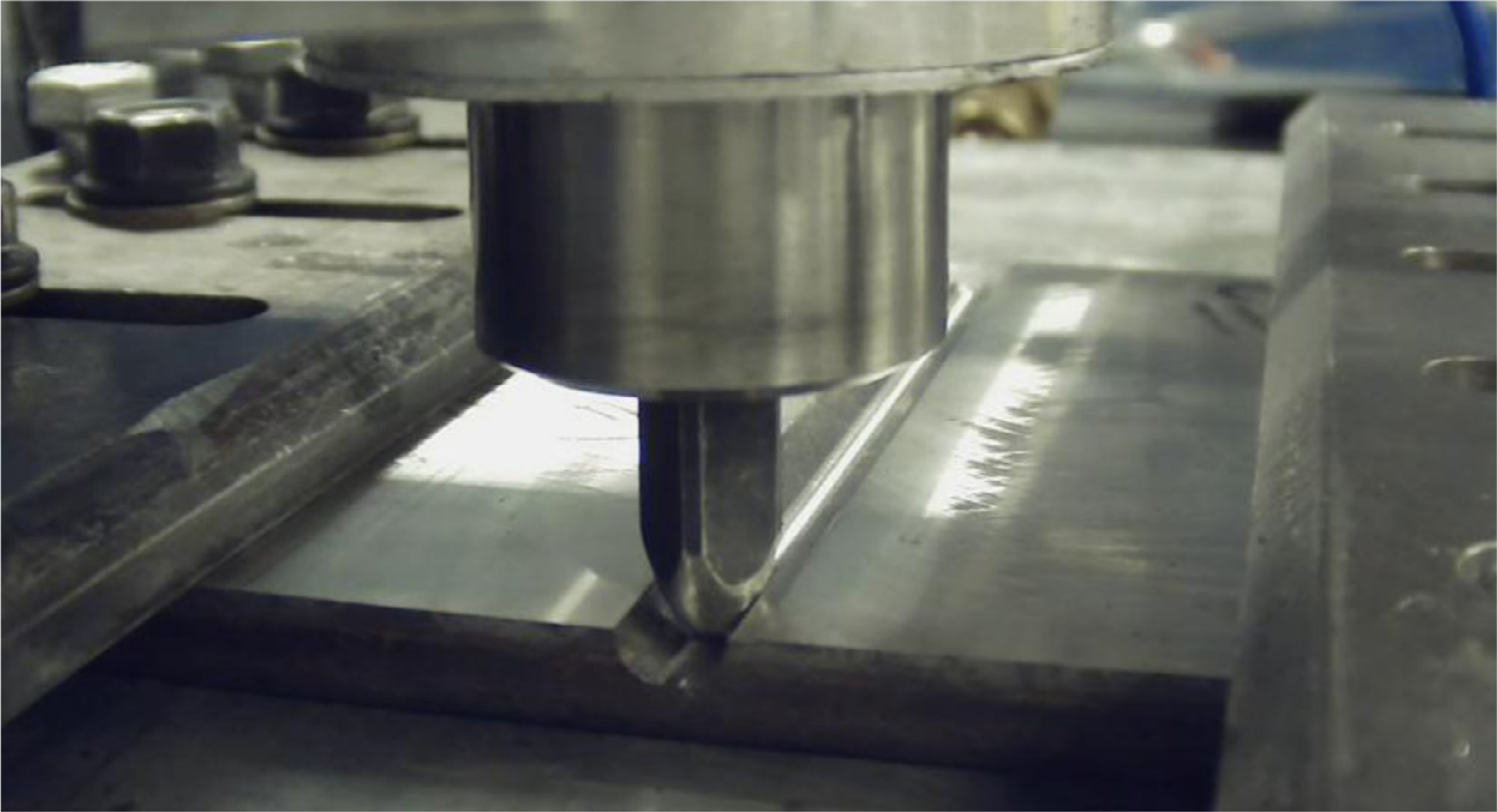
Image of rotating tool, Aermet 100 feedstock material, machined groove “flaw”, and 1080 steel plate substrate during AFSD.

For this dataset, AFSD trials were conducted using a L3 MELD machine to deposit 9.525 mm (0.375 inch) square bar profile Aermet 100 feedstock rods fed through a hollow, 10.16 mm (0.4 inch) diameter rotating tool in the machined flaw region of the 1080 steel plate substrate. Flaw sizes ranged from 4.7625, 6.35, and 9.525 mm depths depending on specific deposition trial and are reported in Table 2. An image of the rotating tool, feedstock material, machined flaw, and substrate are shown in Fig. 6. The investigated deposition parameter conditions for each AFSD trial are presented in Table 2 and are organized into four groups: multi-layer builds deposited at room temperature with and without a cooling plate (MLB), 2-layer builds deposited at room temperature without a cooling plate (TLB), single-layer builds deposited at room temperature without a cooling plate (SLB), and a design of experiments (DOE) for single-layer builds that varied the spindle rotation speed (RPM), travel speed (mm/min), material feed rate (mm/min), and pre-heat temperature (°C) of the deposition. Pre-heated temperatures at 21°C were obtained by performing depositions at room temperature without additional heating. Pre-heated temperatures at 329°C were obtained by performing depositions with a heated backside support anvil with temperature monitored using thermocouples.

**Table 2:** Deposition parameters for ASFD trials of Aermet 100 for a machined flaw region of a 1080 steel plate substrate.

Group Number	Specimen ID	Flaw Size (mm)	Spindle Rotation Speed (RPM)	Tool Traverse Rate on First Pass (mm/min)	Tool Traverse Rate on Subsequent Passes (mm/min)	Filler Bar Feed Rate For First Pass (mm/min)	Filler Bar Feed Rate for Second Pass (IPM)	Cooling Condition	Shoulder Height By Layer (mm)	Pre-heat temperature (°C)
1: Multi-Layer Builds (MLB)	MLD-M1	9.525	400	50.8	203.2	17.78	25.4	Cooling Plate	0.08, 0.58, 1.09, 1.60	21
	MLD-M2	9.525	400	101.6	101.6	91.44	12.7	Cooling Plate	0.13, 0.64	21
	MLD-M3	9.525	350 for Pass 1, 450 for Pass 2	101.6	101.6	91.44	12.7	No Cooling Plate	0, 0.51	21
	MLD-M4	9.525	500	121.92	121.92	109.73	15.24	Cooling Plate	0.13, 0.64	21
	MLD-M5	9.525	500	121.92	121.92	109.73	15.24	No Cooling Plate	0.08, 0.58	21
2: Two-Layer Builds (TLB)	Meld 6	6.35	500	60.96	60.96	30.48	9.14	No Cooling plate	0.51	21
	Meld 7	4.7625	500	60.96	60.96	30.48	9.14	No Cooling plate	0.51	21
3: Single-Layer Builds (SLB)	Meld 8	4.7625	500	38.1	-	25.4	-	No Cooling Plate	-	21
	Meld 9	4.7625	500	60.96	-	25.4	-	No Cooling Plate	-	21
	Meld 10	4.7625	500	38.1	-	30.48	-	No Cooling Plate	-	21
	Meld 11	4.7625	600	60.96	-	30.48	-	No Cooling Plate	-	21
	Meld 12	4.7625	600	60.96	-	25.4	-	No Cooling Plate	-	21
	Meld 13	4.7625	600	38.1	-	30.48	-	No Cooling Plate	-	21
4: Design of Experiments (DOE)	M1-1	4.7625	600	38.1	-	25.4	-	No Cooling Plate	-	21
	M2-1	4.7625	500	60.96	-	30.48	-	No Cooling Plate	-	21
	M3-1	4.7625	600	60.96	-	25.4	-	No Cooling Plate	-	21
	M4-1	4.7625	500	38.1	-	30.48	-	No Cooling Plate	-	21
	M5-1	4.7625	600	60.96	-	30.48	-	No Cooling Plate	-	21
	M6-1	4.7625	500	38.1	-	25.4	-	No Cooling Plate	-	21
	M7-1	4.7625	475	38.1	-	30.48	-	No Cooling Plate	-	329
	M8-2-1	4.7625	375	60.96	-	25.4	-	No Cooling Plate	-	329
	M9-1	4.7625	475	60.96	-	30.48	-	No Cooling Plate	-	329
	M10-1	4.7625	375	38.1	-	25.4	-	No Cooling Plate	-	329
	M11-1	4.7625	475	60.96	-	25.4	-	No Cooling Plate	-	329
	M12-1	4.7625	375	38.1	-	30.48	-	No Cooling Plate	-	329

Metallographic cross-sectioned samples of the as-deposited plates were prepared by cross-sectioning using band saw, mounting in phenolic resin, step-wise grinding with SiC papers, final polishing with 0.05 μm colloidal silica, and etching using 2% Nital etch. Optical images of the polished and etched cross-sections were acquired using an optical microscope with Leco Olympus DP27 macro camera. For each deposition parameter condition, the depth of the heat affected zone for the as-deposited region was measured from optical images starting from the top edge of the 1080 steel plate substrate to the bottom edge of the heat affected zone using ImageJ image processing program. The reported depth of the heat affected zone is an average of five individual measurements. Vickers hardness line traces were collected for samples in Group 2 and Group 4 along the depth of the deposit and heat affected zone extending into the 1080 steel plate substrate on cross-sectioned samples using a Leco LM247AT microhardness tester with a 1000 gf (9.81 N) load and 13 second dwell time. Hardness line traces had a with a 0.50 mm spacing between indents for a total length up to 20 mm. The peak Vickers hardness in the heat affected zone is reported as the maximum measured hardness value in the deformed region of the base metal outside of the deposited Aermet 100.

## Ethics Statement

Not applicable

## CRediT authorship contribution statement

**Kathleen Chou:** Data curation, Writing – original draft. **Michael Eff:** Conceptualization, Investigation, Writing – review & editing. **Chase D. Cox:** Conceptualization, Investigation, Writing – review & editing. **Connor Saukas:** Conceptualization, Investigation, Project administration, Supervision. **Jason Carroll:** Supervision, Funding acquisition.

## Declaration of Competing Interest

The authors declare that they have no known competing financial interests or personal relationships that could have appeared to influence the work reported in this paper.

## Appendix Supplementary Materials

Image, application 1
