# Supplementary figures and images for: Optical image and Vickers hardness dataset for repair of 1080 steel using additive friction stir deposition of Aermet 100
Source: Data Brief. 2022 Jan 22;41:107862. doi: 10.1016/j.dib.2022.107862 (PMC8814329; doi:10.1016/j.dib.2022.107862)

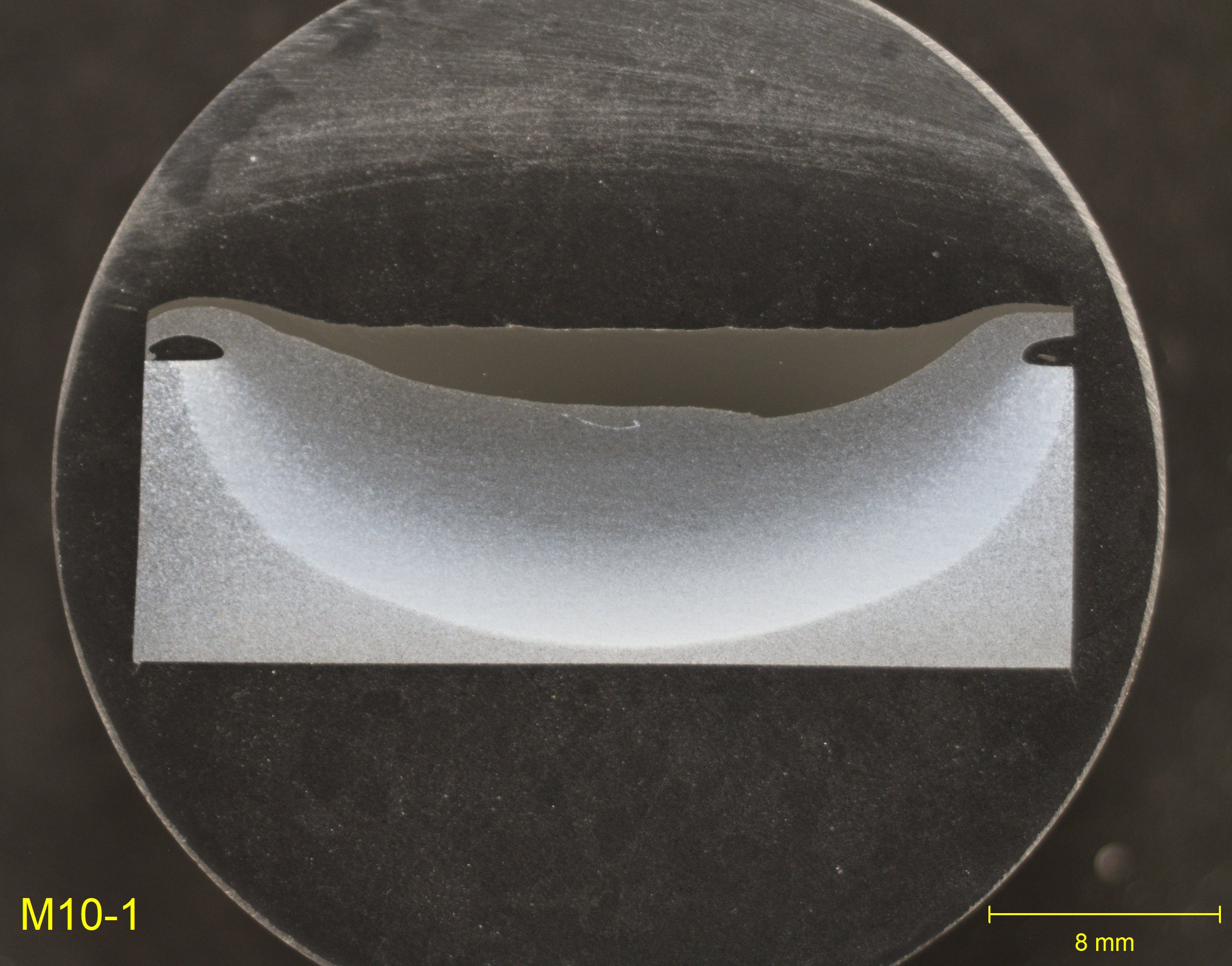

Supplement: Supplementary file 1 [file mmc1.zip › Optical Images of Cross Section/M10-1-Macro.jpg]

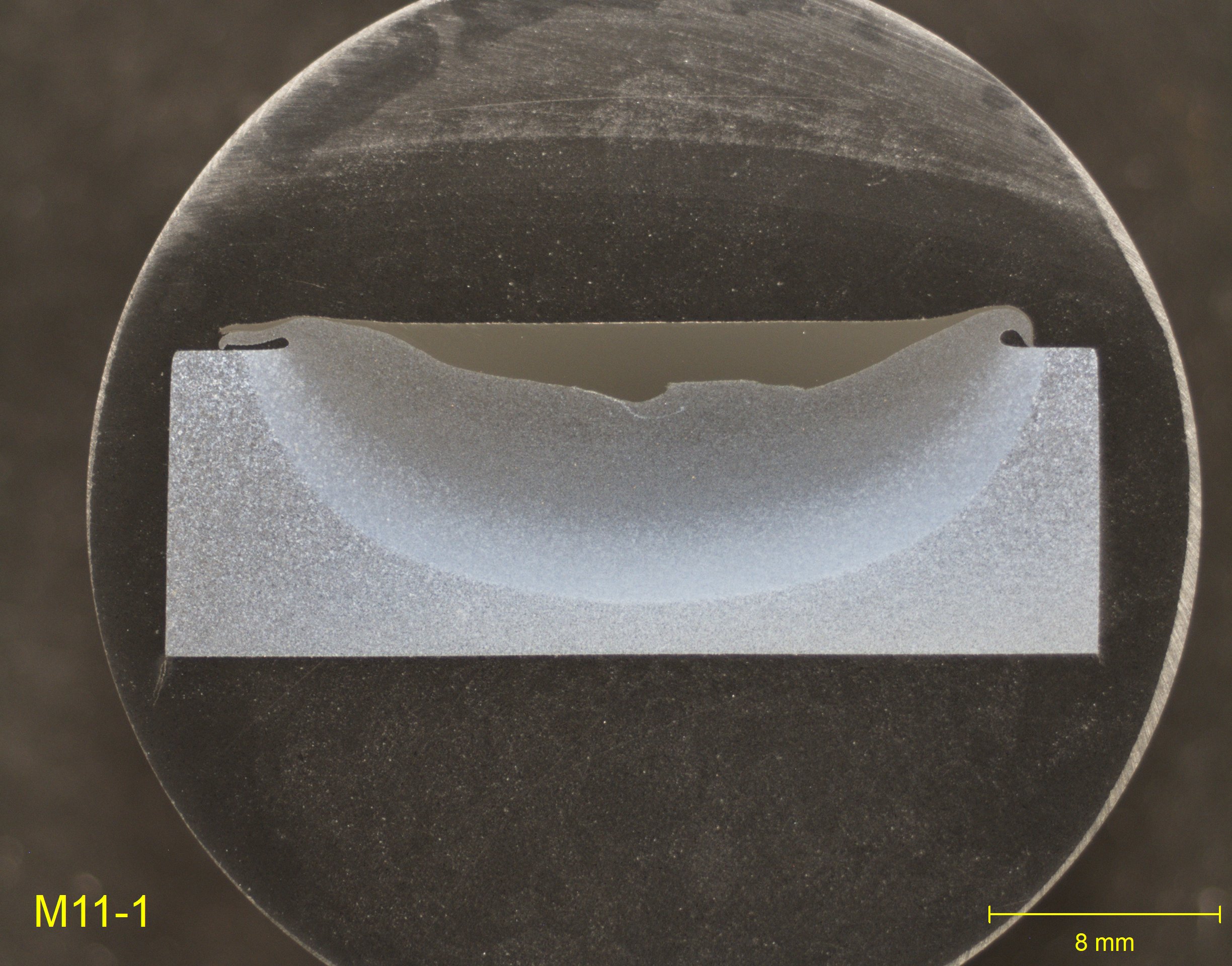

Supplement: Supplementary file 1 [file mmc1.zip › Optical Images of Cross Section/M11-1-Macro.jpg]

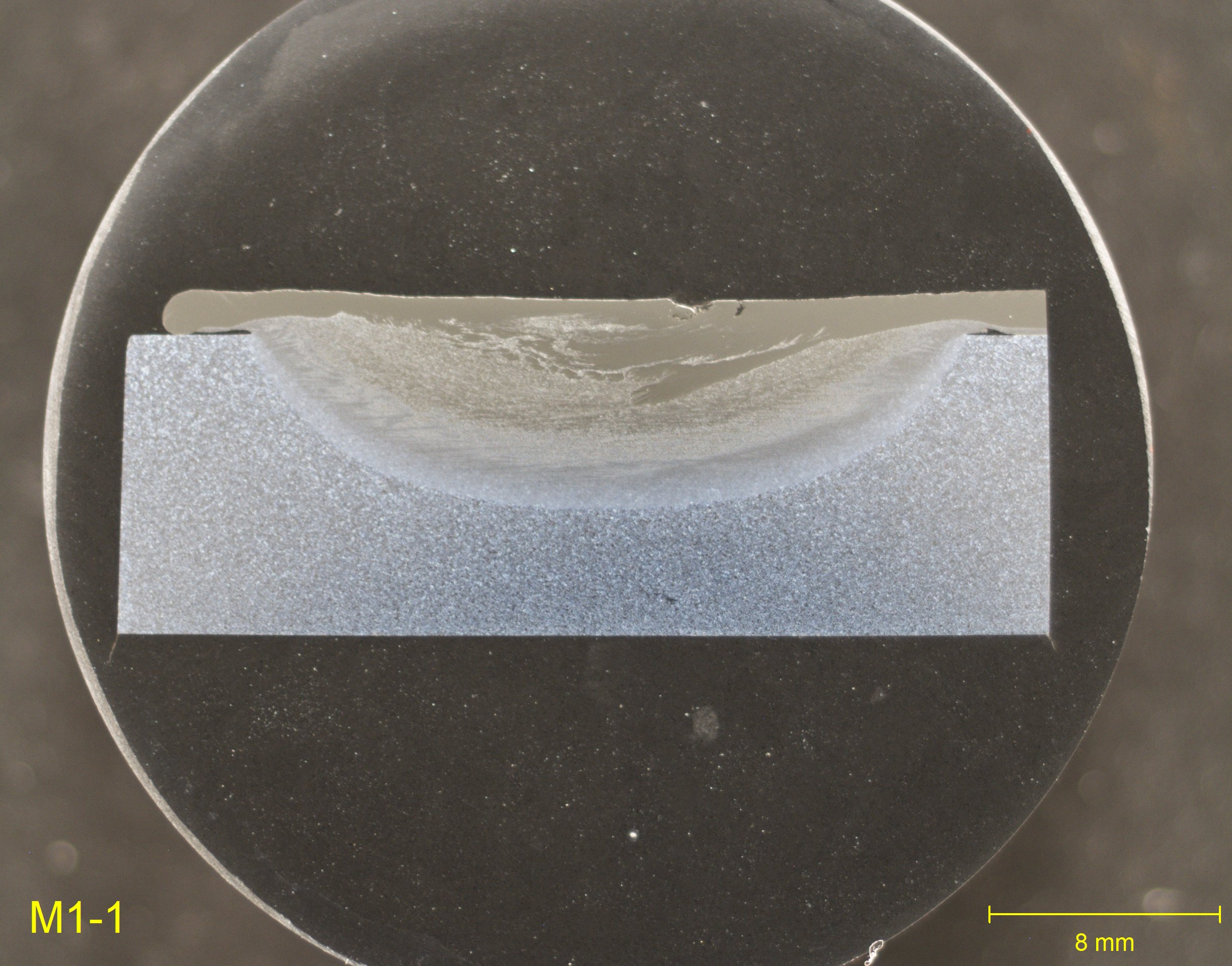

Supplement: Supplementary file 1 [file mmc1.zip › Optical Images of Cross Section/M1-1-Macro.jpg]

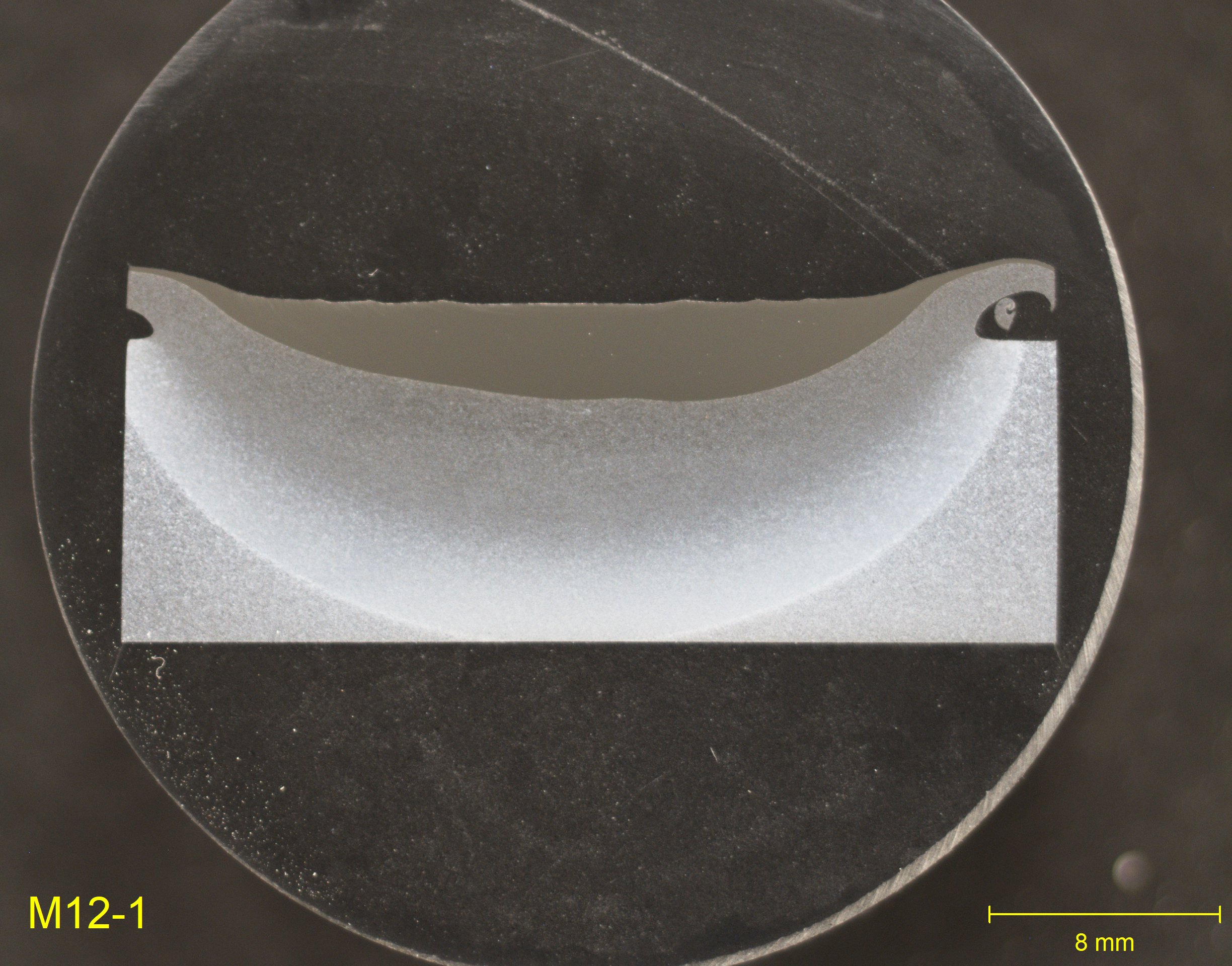

Supplement: Supplementary file 1 [file mmc1.zip › Optical Images of Cross Section/M12-1-Macro.jpg]

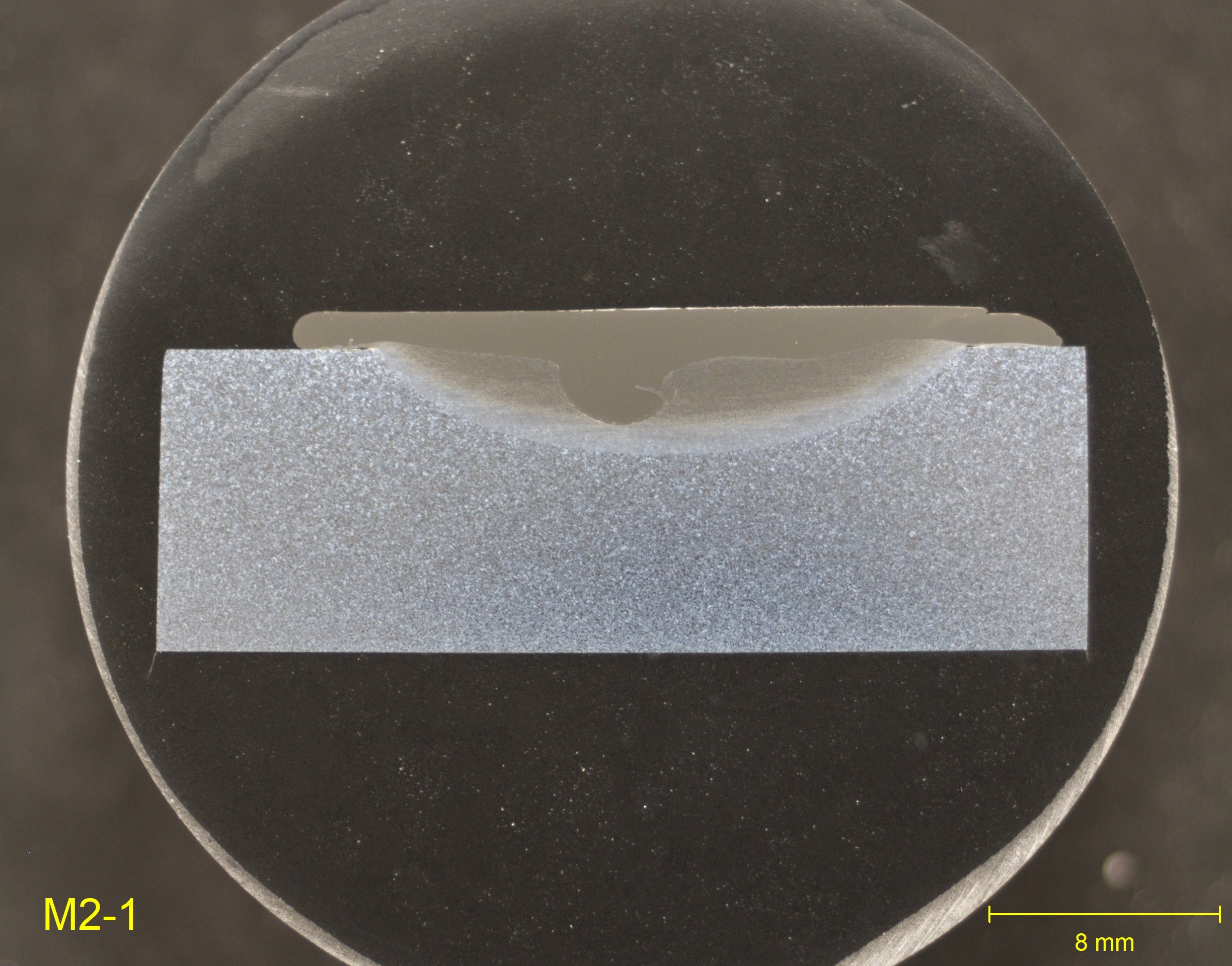

Supplement: Supplementary file 1 [file mmc1.zip › Optical Images of Cross Section/M2-1-Macro.jpg]

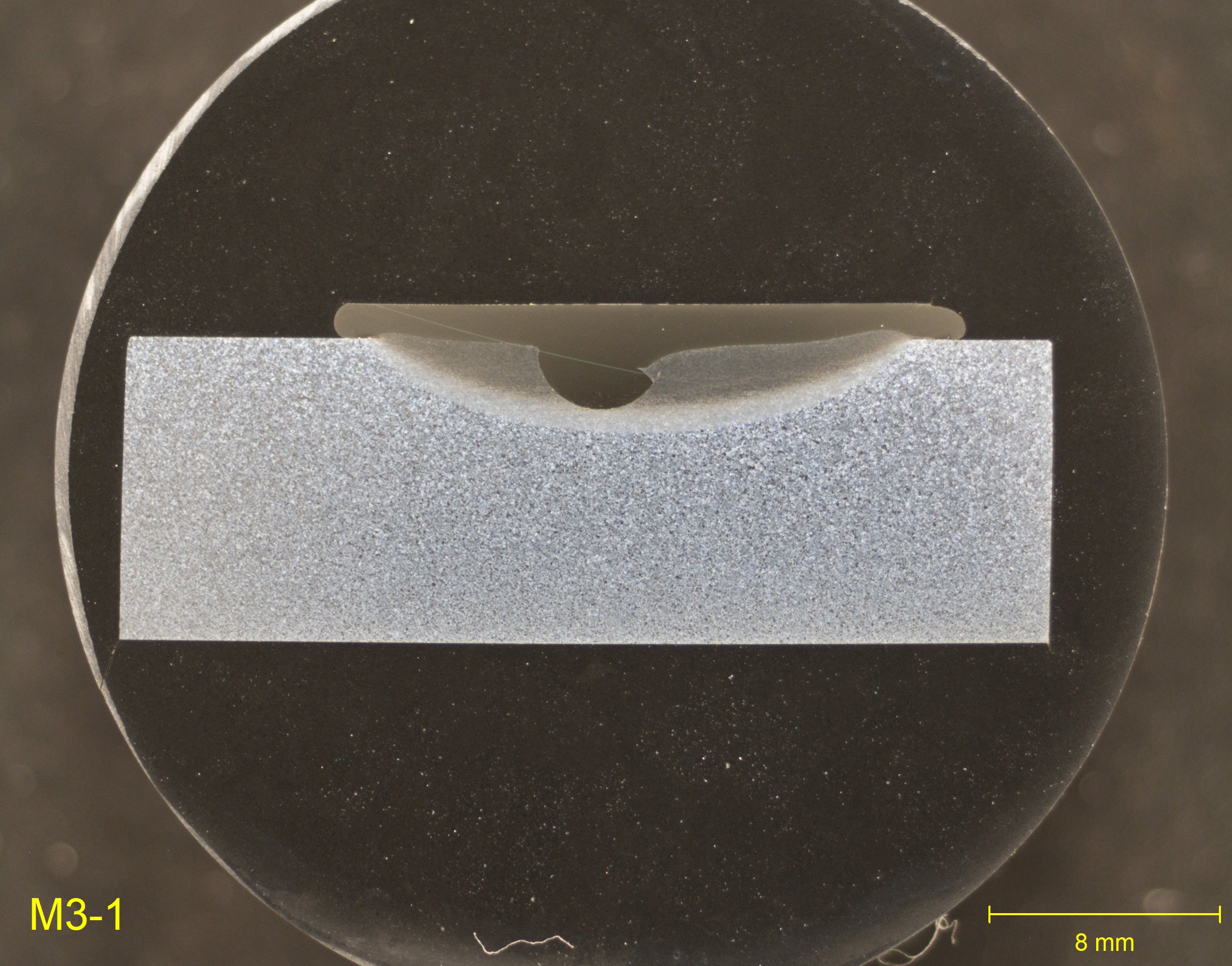

Supplement: Supplementary file 1 [file mmc1.zip › Optical Images of Cross Section/M3-1-Macro.jpg]

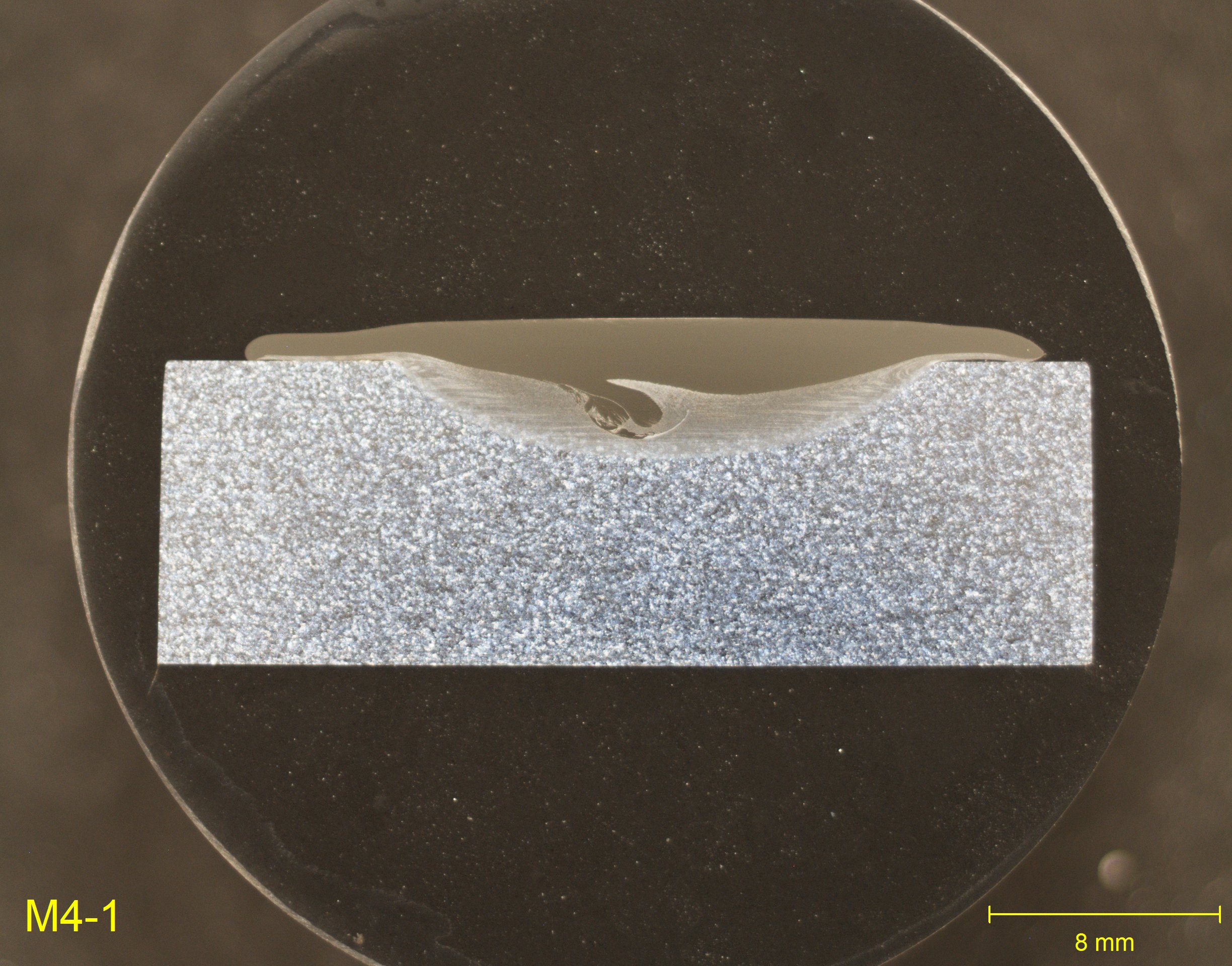

Supplement: Supplementary file 1 [file mmc1.zip › Optical Images of Cross Section/M4-1-Macro.jpg]

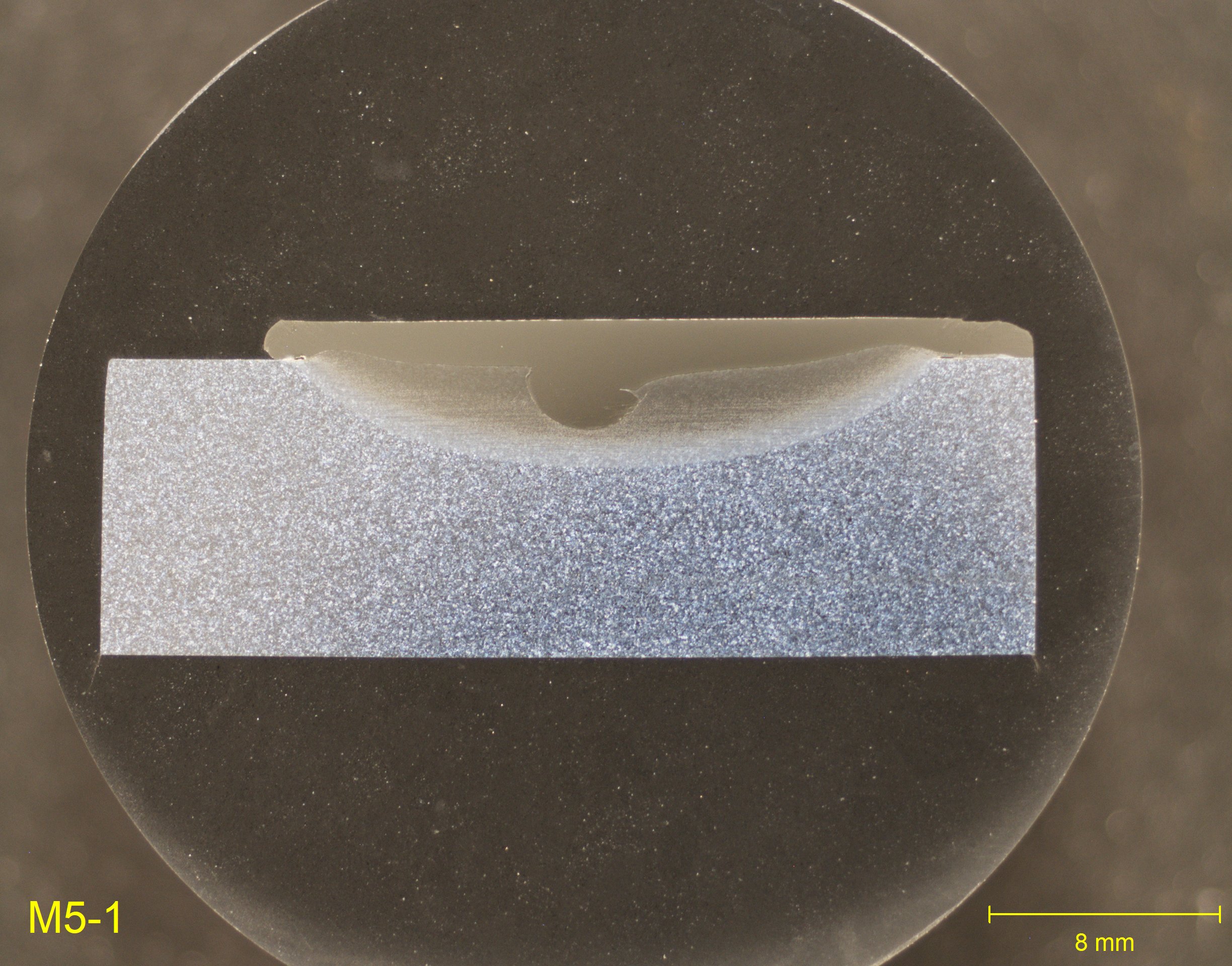

Supplement: Supplementary file 1 [file mmc1.zip › Optical Images of Cross Section/M5-1-Macro.jpg]

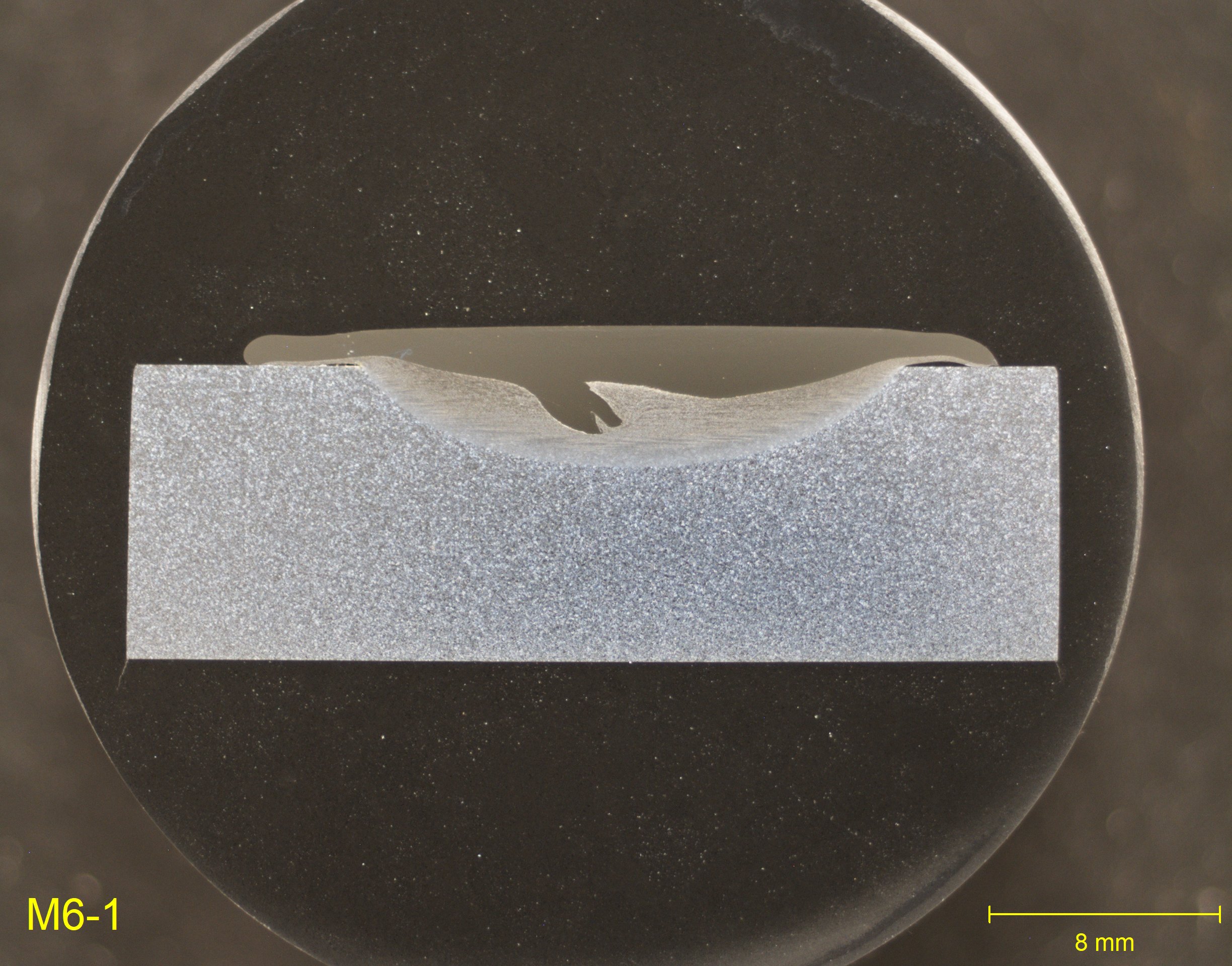

Supplement: Supplementary file 1 [file mmc1.zip › Optical Images of Cross Section/M6-1-Macro.jpg]

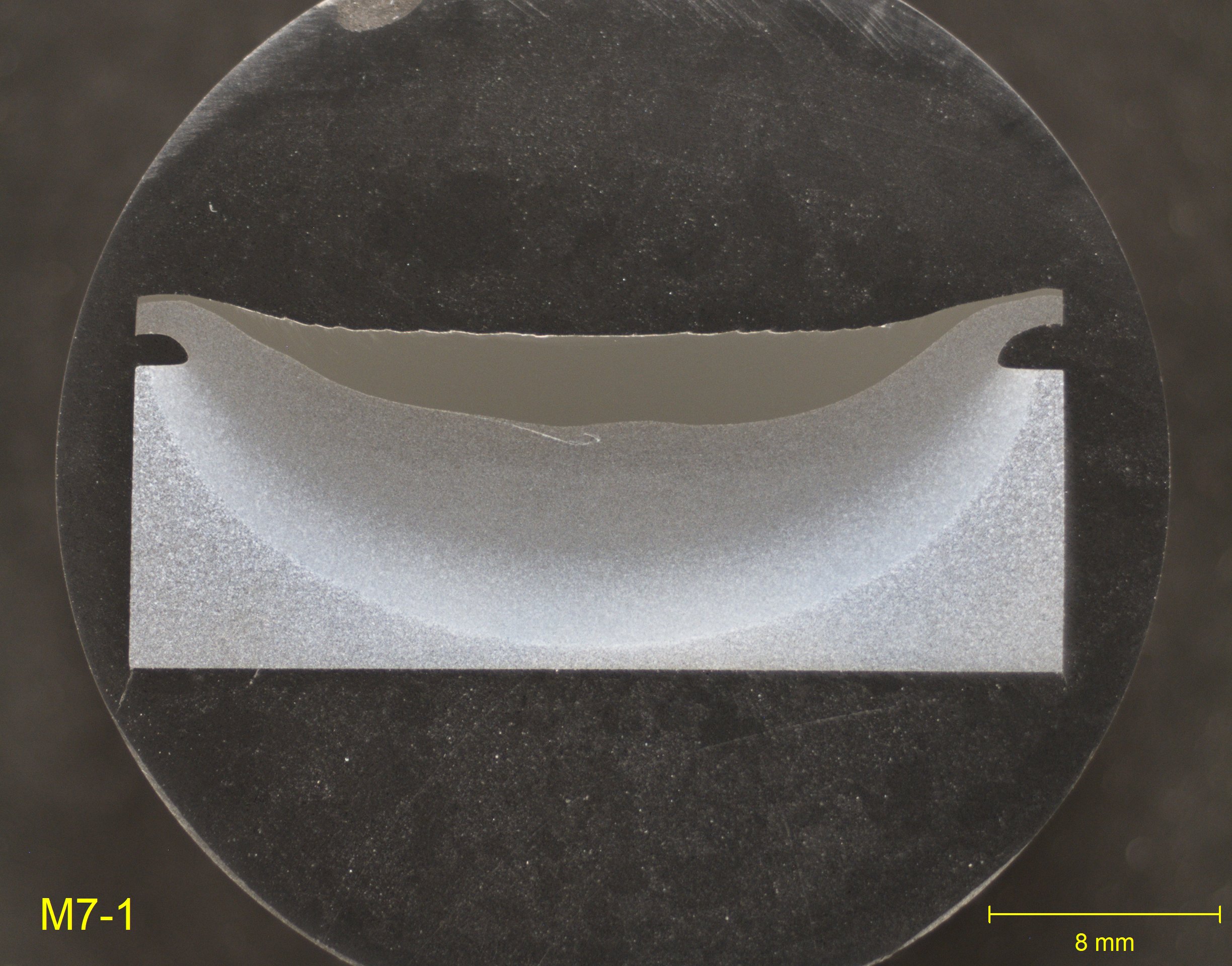

Supplement: Supplementary file 1 [file mmc1.zip › Optical Images of Cross Section/M7-1-Macro.jpg]

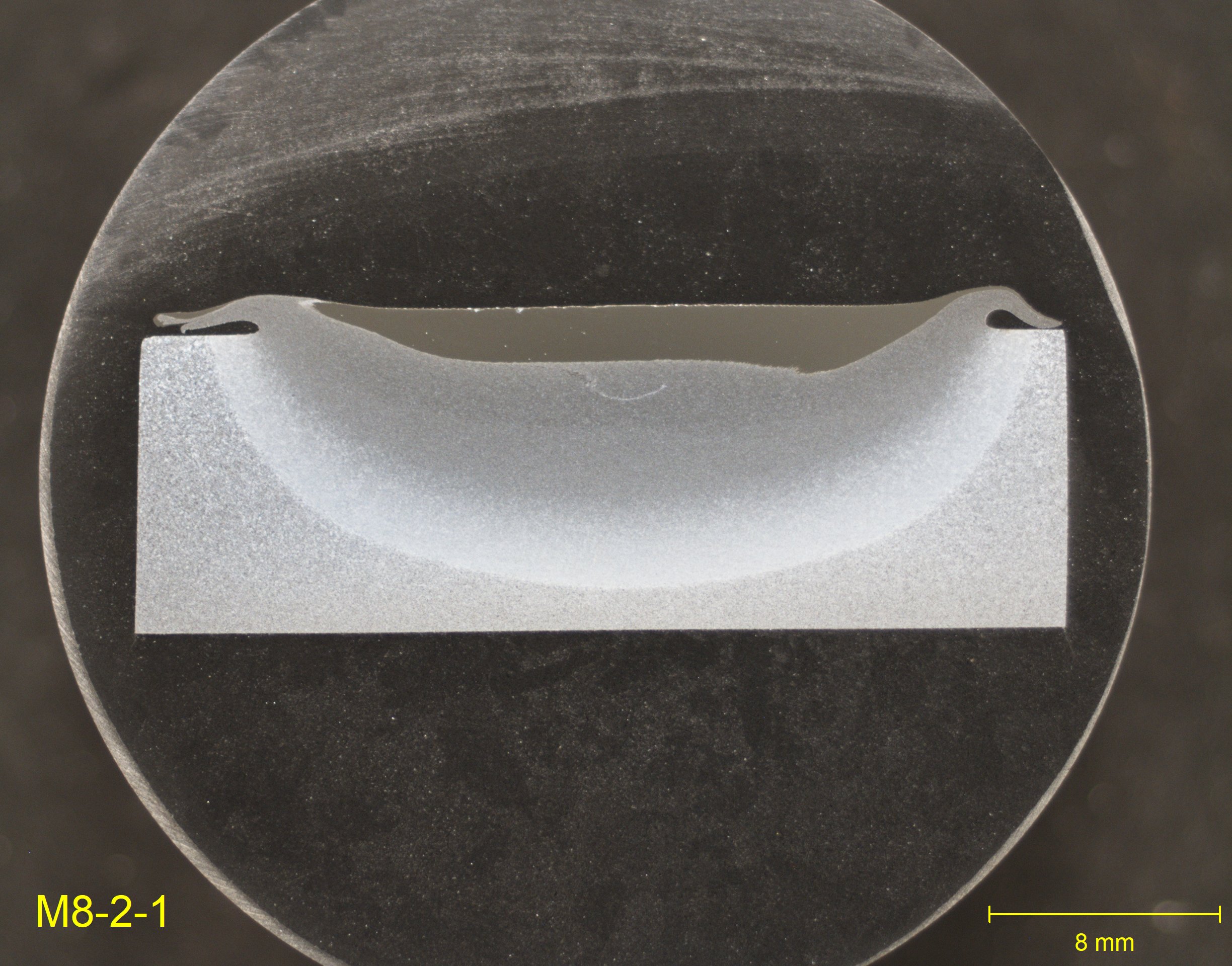

Supplement: Supplementary file 1 [file mmc1.zip › Optical Images of Cross Section/M8-2-1-Macro.jpg]

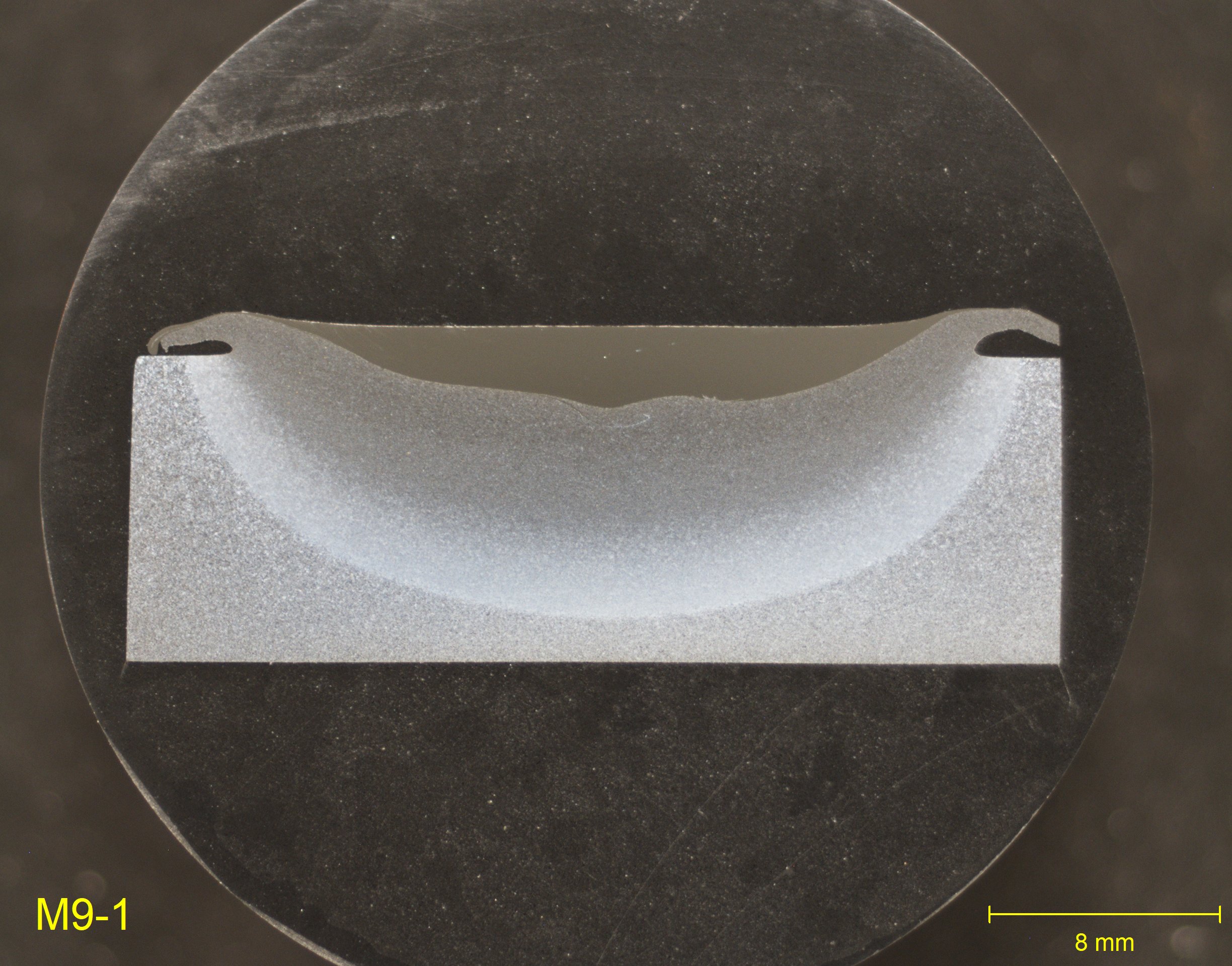

Supplement: Supplementary file 1 [file mmc1.zip › Optical Images of Cross Section/M9-1-Macro.jpg]

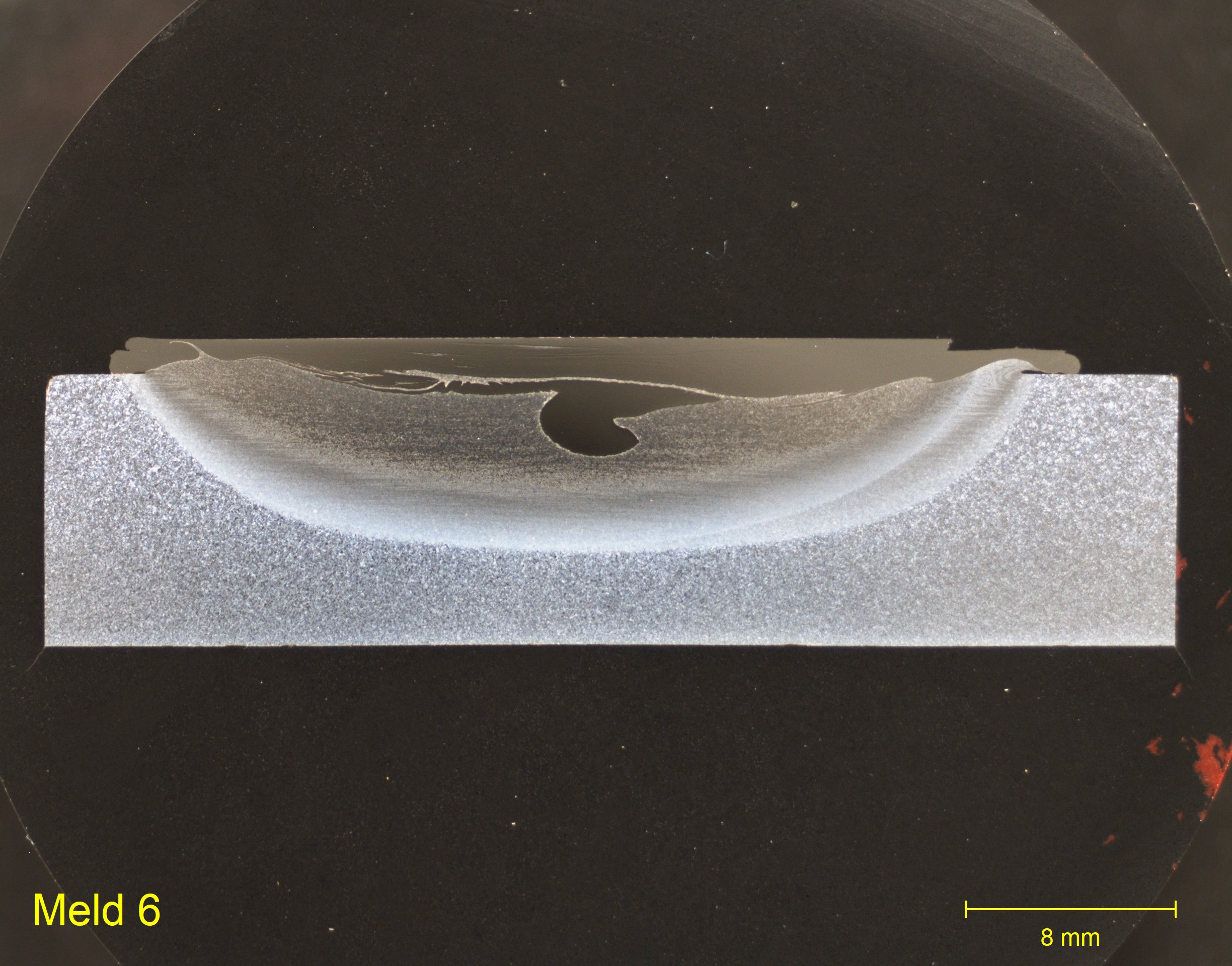

Supplement: Supplementary file 1 [file mmc1.zip › Optical Images of Cross Section/Meld 6-Macro.jpg]

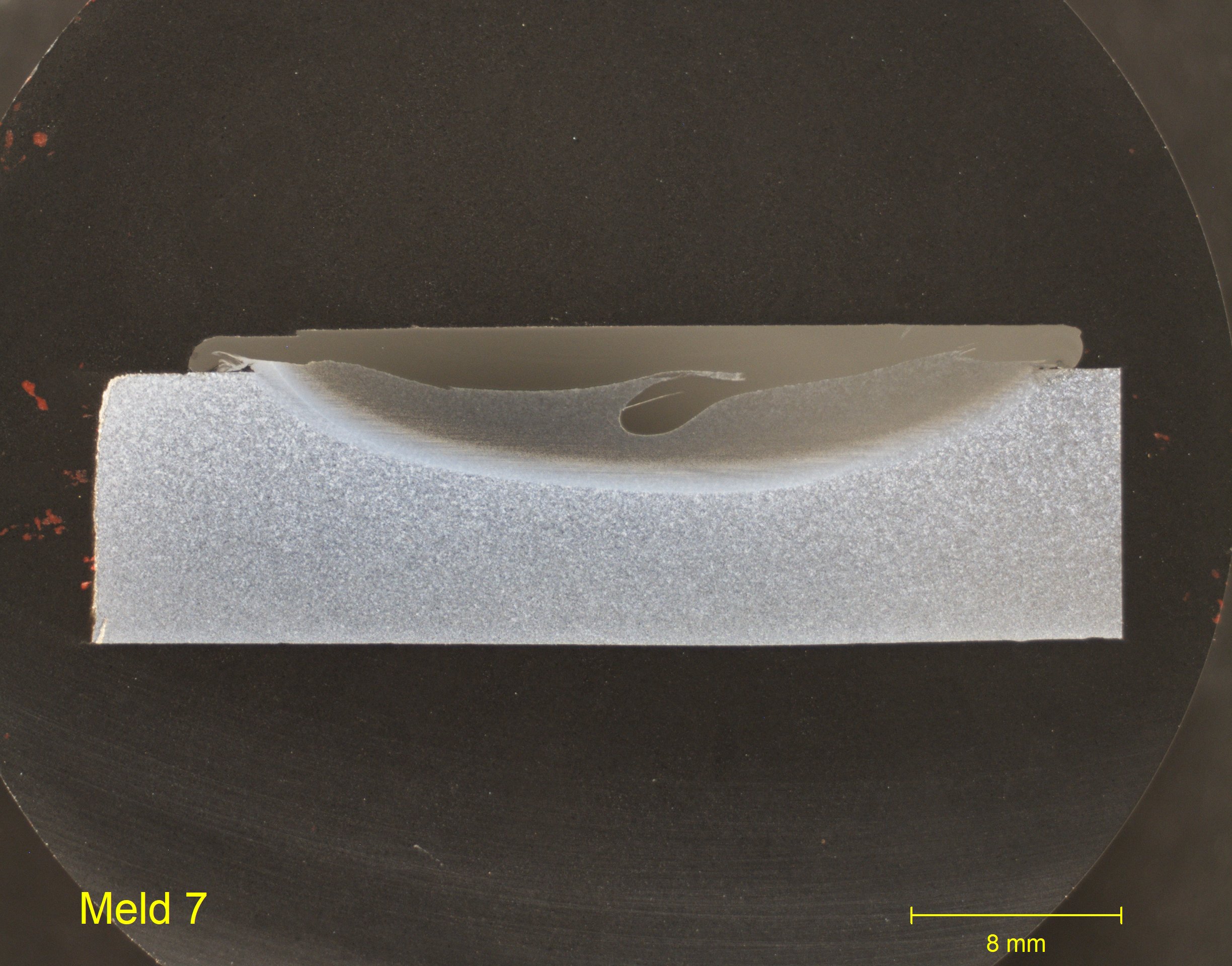

Supplement: Supplementary file 1 [file mmc1.zip › Optical Images of Cross Section/Meld 7-Macro.jpg]

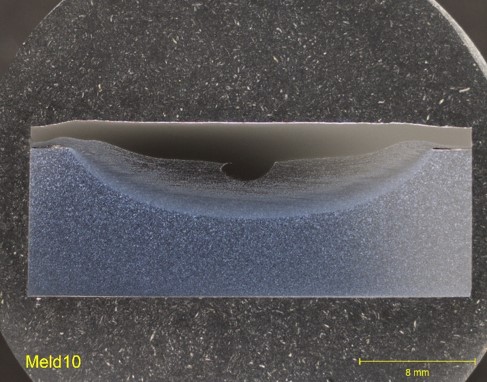

Supplement: Supplementary file 1 [file mmc1.zip › Optical Images of Cross Section/MELD10 Optical Image.jpg]

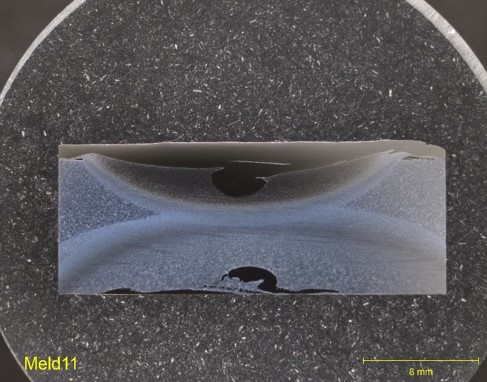

Supplement: Supplementary file 1 [file mmc1.zip › Optical Images of Cross Section/MELD11 Optical Image.jpg]

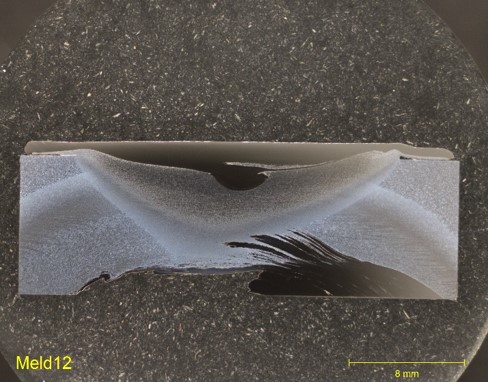

Supplement: Supplementary file 1 [file mmc1.zip › Optical Images of Cross Section/MELD12 Optical Image.jpg]

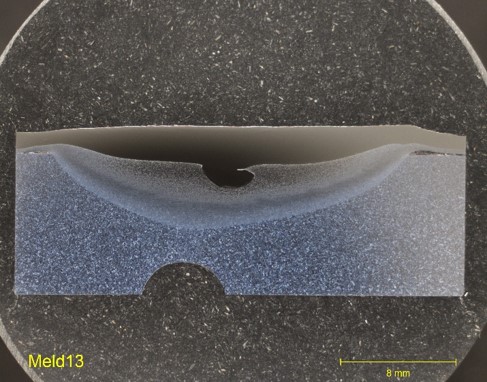

Supplement: Supplementary file 1 [file mmc1.zip › Optical Images of Cross Section/MELD13 Optical Image.jpg]

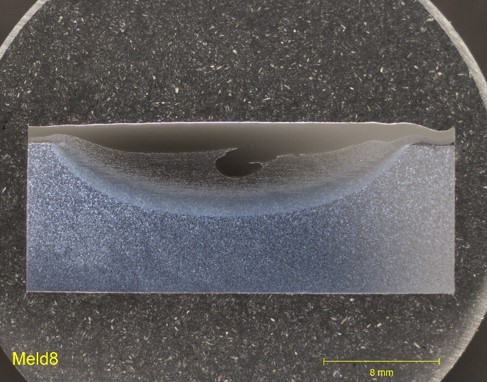

Supplement: Supplementary file 1 [file mmc1.zip › Optical Images of Cross Section/MELD8 Optical Image.jpg]

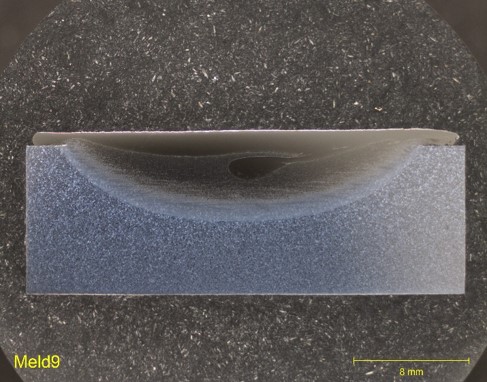

Supplement: Supplementary file 1 [file mmc1.zip › Optical Images of Cross Section/MELD9 Optical Image.jpg]

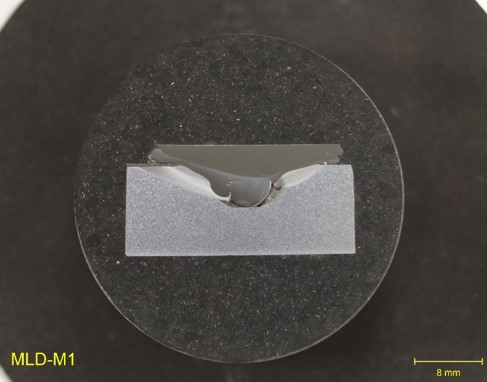

Supplement: Supplementary file 1 [file mmc1.zip › Optical Images of Cross Section/MLD-M1 Optical Image.jpg]

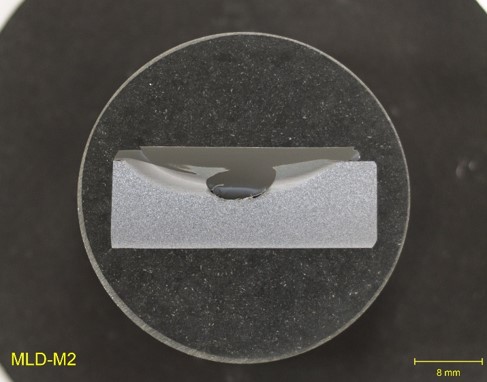

Supplement: Supplementary file 1 [file mmc1.zip › Optical Images of Cross Section/MLD-M2 Optical Image.jpg]

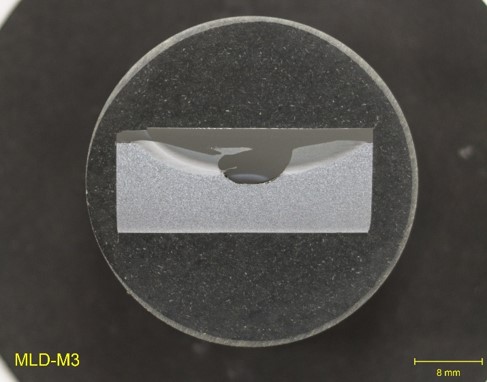

Supplement: Supplementary file 1 [file mmc1.zip › Optical Images of Cross Section/MLD-M3 Optical Image.jpg]

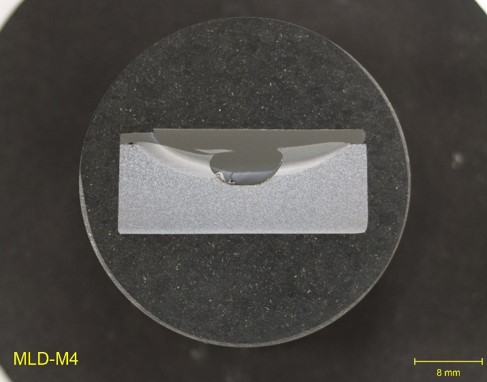

Supplement: Supplementary file 1 [file mmc1.zip › Optical Images of Cross Section/MLD-M4 Optical Image.jpg]

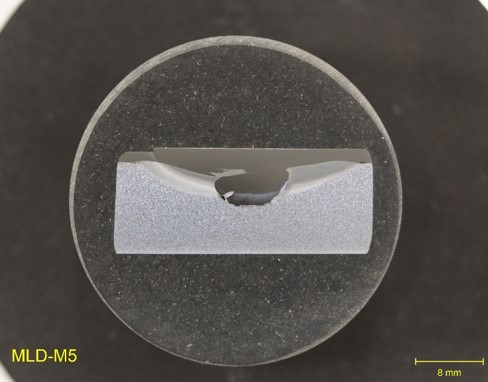

Supplement: Supplementary file 1 [file mmc1.zip › Optical Images of Cross Section/MLD-M5 Optical Image.jpg]
